# Comparison of the Effect of Landmark-Based Midline and Paramedian Approaches on Spinal Anesthesia-Related Complications in Adult Patients: A Meta-Analysis of Randomized Controlled Trials

**DOI:** 10.3390/medicina60010178

**Published:** 2024-01-19

**Authors:** Su Yeon Kim, Hyo-Seok Na, Ji In Park, Keum-O Lee, Hyun-Jung Shin

**Affiliations:** 1 Department of Anesthesiology and Pain Medicine, Seoul National University Bundang Hospital, Seongnam 13620, Republic of Korea; kittyrockz@nate.com (S.Y.K.); hsknana@gmail.com (H.-S.N.); jin@snubh.org (J.I.P.); lko0861@hotmail.com (K.-O.L.); 2 Department of Anesthesiology and Pain Medicine, Seoul National University College of Medicine, Seoul 03080, Republic of Korea

**Keywords:** anesthesia, spinal, back pain, low, meta-analysis, post-dural puncture headache, postoperative complication, postoperative pain, symptom, neurologic

## Abstract

*Background and Objectives:* Spinal anesthesia is widely used in various types of surgery. However, several complications can occur afterward. This study aimed to identify differences in the incidence of anesthesia-related complications according to the approach methods (midline versus paramedian) for landmark-based spinal anesthesia. *Materials and Methods:* We searched electronic databases, including PubMed, EMBASE, CENTRAL, Scopus, and Web of Science, for eligible randomized controlled trials. The primary outcome was post-dural puncture headache (PDPH) incidence, and secondary outcomes were low back pain (LBP) incidence and success rate in the first trial of spinal anesthesia. We estimated the odds ratio (OR) with 95% confidence intervals (CI) using a random-effects model. *Results:* In total, 2280 patients from 13 randomized controlled trials were included in the final analysis. The incidence rates of PDPH were 5.9% and 10.4% in the paramedian and midline approach groups, respectively. The pooled effect size revealed that the incidence of PDPH (OR: 0.43, 95% CI [0.22–0.83]; *p* = 0.01; I^2^ = 53%) and LBP (OR: 0.27, 95% CI [0.16–0.44]; *p* < 0.001; I^2^ = 16%) decreased, and the success rate in the first attempt was higher (OR: 2.30, 95% CI [1.36–3.87]; *p* = 0.002; I^2^ = 35%) with the paramedian than with the midline approach. *Conclusions:* Paramedian spinal anesthesia reduced PDPH and LBP and increased the success rate of the first attempt.

## 1. Introduction

Spinal anesthesia is a type of regional anesthesia commonly used in medical procedures. It involves injecting anesthetic medication into the cerebrospinal fluid in the lower back, specifically into the subarachnoid space surrounding the spinal cord. Spinal anesthesia has been used since it was first performed on pregnant women in 1898 [1]. It temporarily numbs the nerves that transmit sensation from the lower part of the body, resulting in a loss of feeling and muscle paralysis in the lower half. It has been widely employed in various types of surgeries for lesions of the lower abdomen and extremities, such as cesarean section, orthopedic procedures, and urological surgeries. Spinal anesthesia has several benefits, such as reduced re-admission, need for transfusion, surgical site infections, and length of hospital stay compared with general anesthesia; nevertheless, debates continue [2]. Notably, spinal anesthesia may benefit patients with severe comorbidities, impaired respiratory function, or a difficult airway [3].

Two main landmark-based techniques are used to induce spinal anesthesia. The first is the median or midline approach, in which the needle passes through the supraspinous and interspinous ligaments and the ligamentum flavum to pierce the dura [4]. The second is the paramedian approach, which targets the midline, where the needle insertion point starts slightly inferolateral to it. It passes through the ligamentum flavum and pierces the dura, evading the supraspinous and interspinous ligaments [4].

Despite its advantages, spinal anesthesia should be carefully performed to minimize complications. Post-dural puncture headache (PDPH) is one of the most common complaints after lumbar puncture [5]. Headache is caused by leakage and an inadequate replacement of the cerebrospinal fluid (CSF), which decreases intracranial pressure (ICP) [5]. The overall incidence of PDPH after neuraxial intervention is 36% [6]. Low back pain (LBP) is another relatively common complication with an incidence of approximately 6% [7]. Epidural hematoma, transient neurological symptoms, meningitis, urinary retention, nerve injury, and local anesthetic systemic toxicity may also occur [5]. Previous studies have investigated whether the method of spinal anesthesia influences the occurrence of these complications, but often yielded conflicting results [8,9,10,11].

We designed this meta-analysis to determine whether both landmark-based approaches for spinal anesthesia (midline and paramedian) affect post-induction PDPH and LBP incidence. We also compared their success rates in one attempt.

## 2. Materials and Methods

This meta-analysis followed the Preferred Reporting Items for Systematic Reviews and Meta-Analyses (PRISMA) statement after registering the predefined study protocol in the International Prospective Register of Systematic Reviews (PROSPERO; identifier: CRD42023399503) [12].

### 2.1. Eligible Criteria

Eligible studies were selected using the following criteria: (1) randomized clinical trials (RCTs); (2) spinal anesthesia in adults using the landmark technique; and (3) studies that compared midline and paramedian approaches for spinal anesthesia. However, we excluded ultrasound-guided spinal anesthesia studies, animal studies, case reports, observational studies, retrospective studies, narrative and systematic review articles, study protocols, editorials, letters, and abstract-only articles.

### 2.2. Search Strategy

We searched electronic databases, including PubMed, EMBASE, CENTRAL, Scopus, and Web of Science, for eligible RCTs from their inception to 19 December 2022, without limitations on journal, regions, publication year, or language. The search terms included “median”, “midline”, “paramedian”, “para-median”, “lateral”, “spinal”, “intrathecal”, “intra-thecal”, “subarachnoid”, “sub-arachnoid”, “anesthesia”, “anaesthesia”, and “block”. Supplementary Table S1 lists the literature search strategies.

### 2.3. Study Selection

Two authors (S.Y.K. and H.-J.S.) independently reviewed eligible RCTs for data analysis. Titles and abstracts were used to screen the pooled studies from each database. Subsequently, full-text evaluations were performed to select eligible studies. In case of disagreements, we sought the opinions of a third reviewer (H.-S.N.).

### 2.4. Data Extraction

After reviewing the final RCTs, we collected the following data: authors, year of publication, number of participants, surgery type, spinal needle type used, PDPH incidence, LBP incidence, and success rates of the first attempt at spinal anesthesia. The values were extracted from the images using WebPlotDigitizer (https://apps.automeris.io/wpd/; accessed on 13 January 2023) for the data presented as graphs.

### 2.5. Assessment of Risk of Bias

The risk of bias in the included studies was evaluated independently by two reviewers using the revised Cochrane risk-of-bias tool for randomized trial 2 (RoB 2) [13]. RoB 2 assesses studies based on the following six categories: (1) randomization process, (2) deviations from intended interventions, (3) missing outcome data, (4) measurement of the outcome, (5) selection of the reported result, and (6) overall bias. The risk of bias was classified as “low risk,” “some concerns,” or “high risk.”

### 2.6. Certainty of Evidence

Using the Grading of Recommendations, Assessment, Development, and Evaluation (GRADE) system [14], we determined the certainty level of evidence for each outcome. Five domains—risk of bias, inconsistency, indirectness, imprecision, and publication bias—were included in the GRADE system.

### 2.7. Outcome Measures

The primary outcome was incidence of PDPH after spinal anesthesia. Additionally, LBP incidence and success rates of the procedure were secondary outcomes.

### 2.8. Statistical Analyses

Meta-analyses were performed using Review Manager (RevMan, Version 5.4.1, The Cochrane Collaboration, Copenhagen, Denmark). Odds ratios (ORs) were calculated to compare the effect sizes for dichotomous variables. A random-effects model was used owing to the different treatment effect sizes.

Sensitivity analysis identified small-study effects using the leave-one-out method. Cochran’s Q test and I^2^ statistics were used to calculate the heterogeneity levels. Heterogeneity among studies was defined as high (I^2^ = 76–100%), moderate (I^2^ = 26–75%), or low (I^2^ = 0–25%). Publication bias was not assessed because all meta-analyses included fewer than 10 independent studies [15]. Sensitivity analysis was tested using the “metafor” package in R software (version 4.1.3, R Foundation for Statistical Computing, Vienna, Austria). Statistical significance was set at *p* < 0.05.

## 3. Results

### 3.1. Study Selection

**Figure 1:**
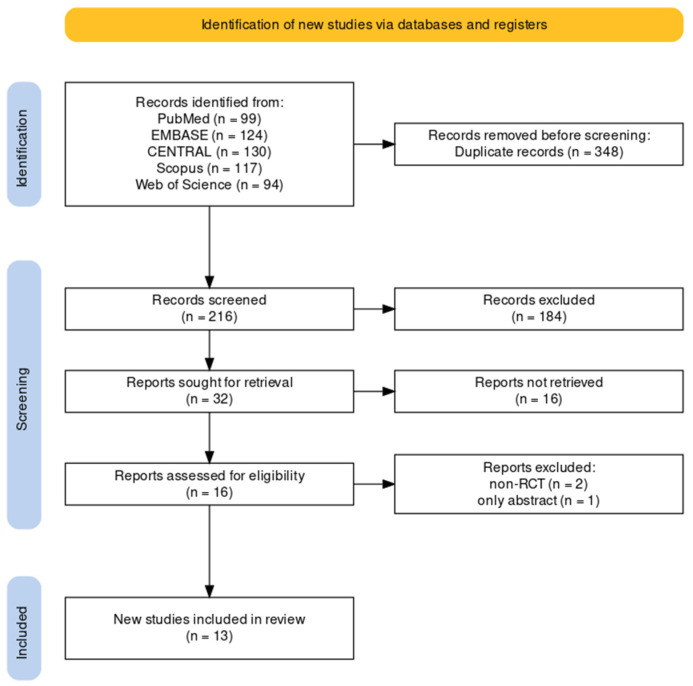
Flow diagram of study selection.

Overall, 564 articles were extracted from the initial searches of PubMed (*n* = 99), EMBASE (*n* = 124), CENTRAL (*n* = 130), Scopus (*n* = 117), and Web of Science (*n* = 94). After removing 348 duplicate articles, 184 and 16 were excluded based on the title and abstract, respectively. The full texts of 16 articles were reviewed for eligibility, and 13 articles were included in the final analysis (Figure 1).

Table 1 summarizes the characteristics of the included articles. In total, 2280 participants were included in this study. Most studies [8,10,11,16,17,18,19,20,21] involved patients undergoing cesarean sections, except four, which included hip fracture surgery [22], lower abdominal surgery [23,24], and “other types of surgery” such as urological, orthopedic, gynecological, and general surgeries [25]. Six studies used cutting needles, such as Quincke [8,10,18,20,24] and Tuohy [22]. However, one study used the Sprotte needle [17], and another used the Whitacre needle [20] or described that the pencil-point needle was used without specifying the spinal needle model [16,25]. The spinal needle gauge ranges from 19 G [22], 23 G [8], and 25 G [10,11,16,18,21,23,24,25] to 27 G [20]. A study [20] compared four approach groups: the Quincke needle midline, Quincke needle paramedian, Whitacre needle midline, and Whitacre needle paramedian. Therefore, we divided this study into two sub-studies based on the spinal needles used in the four groups. Lee et al. [25] did not measure the success rate as an outcome variable; nonetheless, calculating the probability of success on the first attempt was possible because the authors set the exclusion criterion for cases where spinal anesthesia was attempted more than twice and recorded the number of excluded cases. We added the cases excluded due to lost-to-follow-up to those in which spinal anesthesia was successful in the first attempt.

### 3.2. Incidence of Post-Dural Puncture Headache

**Figure 2:**
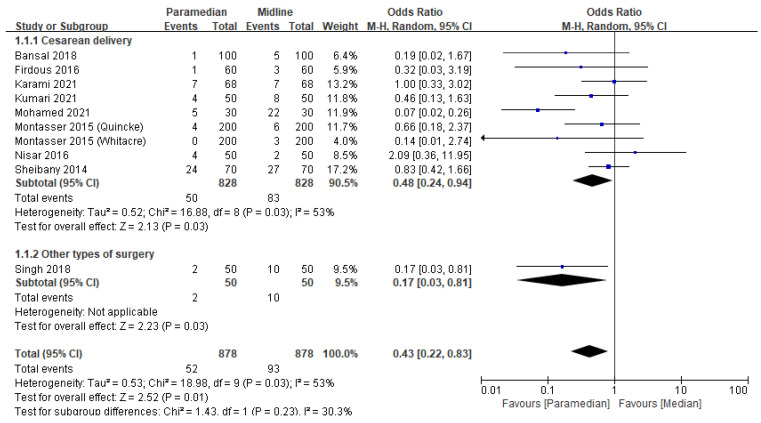
Forest plot: Effect of midline and paramedian approach on post-dural puncture headache. M-H: Mantel–Haenszel [8,10,11,16,17,19,20,21,23].

Nine studies [8,10,11,16,17,19,20,21,23] with 1756 participants were included in this meta-analysis. The incidence of PDPH was 5.9% and 10.6% in the paramedian and midline approach groups, respectively. The pooled effect size revealed that PDPH incidence decreased in the paramedian approach group (OR: 0.43, 95% confidence interval (CI) [0.22–0.83]; *p* = 0.01; I^2^ = 53%) (Figure 2). 

In the subgroup analysis, PDPH incidence was reduced in patients who underwent cesarean delivery (OR: 0.48, 95% CI [0.24–0.94]; *p* = 0.03; I^2^ = 53%) and low abdominal surgery (OR: 0.17, 95% CI [0.03–0.81]; *p* = 0.03; I^2^ = not applicable) when the paramedian approach was used (Figure 2). 

Sensitivity analysis revealed that the pooled effect did not differ significantly (OR: 0.37, 95% CI [0.19–0.73]; *p* = 0.005; I^2^ = 53%) after omitting an outlier [21]. However, interpretation requires caution owing to the moderate degree of heterogeneity.

### 3.3. Incidence of Low Back Pain

**Figure 3:**
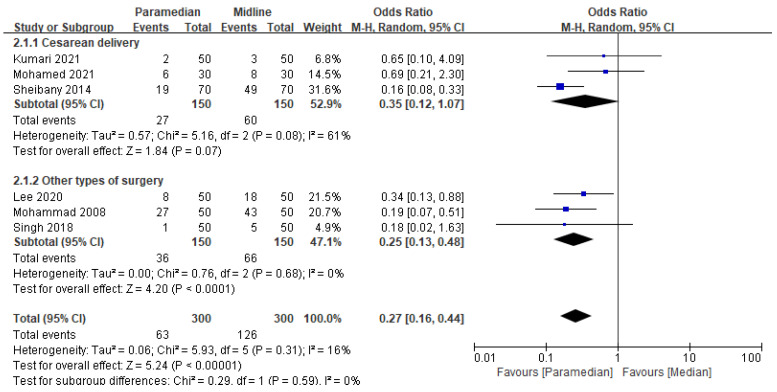
Forest plot: Effect of midline and paramedian approach on low back pain. M-H: Mantel–Haenszel [11,17,19,23,24,25].

LBP incidence was reported in six studies (*n* = 600) [11,17,19,23,24,25]. The paramedian approach significantly reduced LBP incidence (OR: 0.27, 95% CI [0.16–0.44]; *p* < 0.001; I^2^ = 16%) (Figure 3).

Subgroup analysis revealed that the paramedian approach reduced incidence of LBP in participants who underwent “other types of surgery” (OR: 0.25, 95% CI [0.13–0.48]; *p* < 0.001; I^2^ = 0%), whereas not in those who underwent cesarean delivery (OR: 0.35, 95% CI [0.12–1.07]; *p* = 0.07; I^2^ = 61%) (Figure 3).

In the sensitivity analysis, the size and direction of pooled estimates were maintained (OR: 0.32, 95% CI [0.19–0.55]; *p* < 0.001; I^2^ = 0%) after omitting an outlier [11]. 

### 3.4. Success Rate on the First Attempt

**Figure 4:**
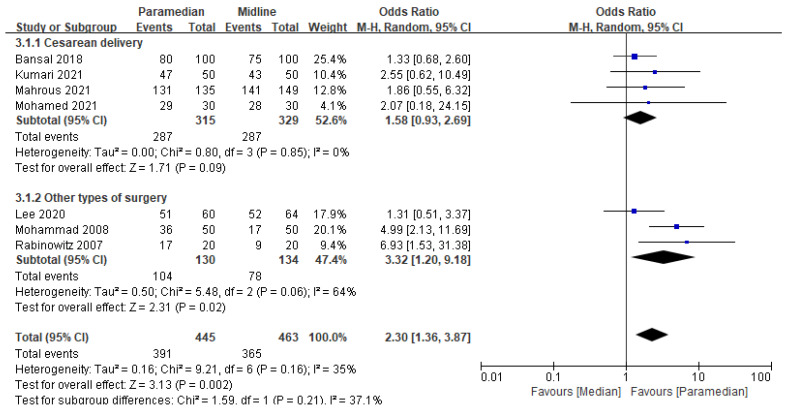
Forest plot: Effect of midline and paramedian approach on success rate on first attempt. M-H: Mantel–Haenszel [8,17,18,19,22,24,25].

Seven RCTs [8,17,18,19,22,24,25] with 908 participants were included in this meta-analysis. Compared to the median approach, the success rate at the first attempt was higher (OR: 2.30, 95% CI [1.36–3.87]; *p* = 0.002; I^2^ = 35%) with the paramedian approach (Figure 4).

However, when subgroup analysis was performed based on the type of surgery, no significant difference was observed between both the paramedian and the midline approaches in the cesarean delivery subgroup (OR: 1.58, 95% CI [0.93–2.69]; *p* = 0.09; I^2^ = 0%) in contrast to the “other types of surgery” subgroup (OR: 3.32, 95% CI [1.20–9.18]; *p* = 0.02; I^2^ = 64%) (Figure 4).

Sensitivity analysis uncovered that the pooled effect size remained (OR: 1.76, 95% CI [1.14–2.71]; *p* = 0.01; I^2^ = 0%) after omitting an outlier [24].

### 3.5. Risk of Bias

**Figure 5:**
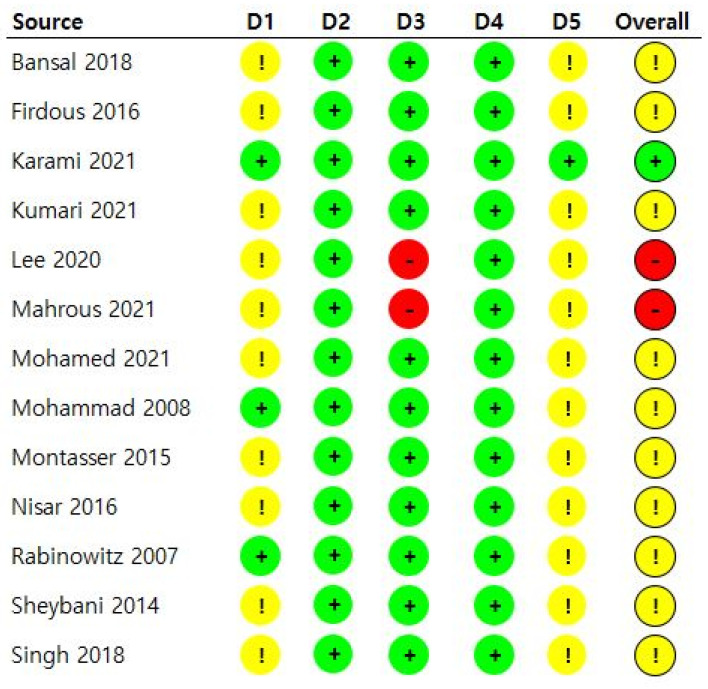
Risk of bias summary. D1, randomization process; D2, deviations from the intended interventions; D3, missing outcome data; D4, measurement of the outcome; D5, selection of the reported result; 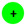, low risk; 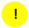, some concerns; 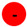, high risk [8,10,11,16,17,18,19,20,21,22,23,24,25].

The overall risk of bias was scored as “low risk” in one study [10], “some concerns” in ten [8,11,16,17,19,20,21,22,23,24], and “high risk” in two studies [18,25]. Two studies [18,25] ranked as “high risk” in the domain of missing outcome data owing to many drop-outs. Most studies [8,11,16,17,18,19,20,21,23,25] did not describe whether the allocation sequence or the details regarding cancellation was concealed, resulting in “some concerns” in the randomization. Only one study [10] matched the RCT registration site protocol and research method conducted. Figure 5 summarizes the risk of bias.

### 3.6. The Certainty Level of the Evidence

The certainty levels for PDPH and LBP incidence were high, whereas the success rate at the first attempt was assessed as moderate. Supplementary Table S2 provides details of the certainty assessment.

## 4. Discussion

This meta-analysis revealed that the paramedian approach for spinal anesthesia decreased the incidence of PDPH and LBP and increased the success rate at first attempt compared to the midline technique.

The mechanism of PDPH is unclear; nevertheless, CSF leakage via the dural hole caused by needle passage is traditionally considered a key factor [26]. PDPH is a type of dull headache that typically occurs after a lumbar puncture. It is characterized by worsening when sitting or standing and improves when lying down [6]. PDPH typically occurs within 48 h following a lumbar puncture. While most cases resolve naturally within 2–3 days, some patients may experience lingering symptoms even after discharge [27]. Although the majority of cases involve only headaches without additional complications, CSF leakage leading to reduced ICP can result in the drooping and rupture of bridging veins [27]. In severe cases, this can lead to the development of an intracranial subdural hematoma, emphasizing the importance of vigilance and monitoring for potential complications. Treatment options of PDPH include bed rest, hydration or injection of intravenous fluids, and caffeine, although their efficacy is often limited [6]. Symptomatic relief may also be attempted using non-steroidal anti-inflammatory drugs (NSAIDs) or acetaminophen [27]. The gold standard for treating PDPH is an epidural blood patch [6]. This procedure involves injecting the patient’s own blood into the epidural space to seal any leakage of the CSF. However, epidural blood patch is contraindicated in cases such as infection at the injection site or coagulopathy. In such instances, where contraindications are present, conservative management focusing on symptomatic relief is often pursued as PDPH tends to naturally resolve over time. Continuous efforts have been made to prevent PDPH, such as selecting a pencil-point and smaller needle diameter [26], to minimize CSF leakage. Our findings suggest that the approach could be a modifiable factor in reducing PDPH incidence, which was lower with the paramedian than the midline technique.

Several hypotheses have supported the role of this approach in reducing PDPH incidence. The first mechanism is the flap valve mechanism. When the needle advances in the paramedian approach, a smaller entry angle is required than in the midline technique, resulting in an oblique needle track on the thick dura mater. This makes the opening site of the dura mater resemble a flap valve that closes [28]. Additionally, the different penetration sites on the dural and arachnoid layers, which do not overlap, produce a second flap valve function [28]. Second, a self-blood patching effect was observed through the internal vertebral venous plexus puncture in the epidural space. The venous plexus is more abundant in the anterolateral and posterolateral epidural spaces than in the midline area [29]. The anatomical location of the venous plexus can increase the likelihood of passing the needle through the paramedian approach. Blood clots in the epidural space can act as a blood patch. This process should not be considered a procedural complication but rather a normal process that can occur during spinal anesthesia. Finally, less trauma to the dura mater from fewer injection attempts may reduce the CSF leakage.

LBP is a common acute complication of spinal anesthesia and is defined as localized pain at the puncture site without significant neurological symptoms, such as radiating pain to the lower extremities [30,31]. The difference in LBP incidence between the paramedian and midline approaches may be due to the anatomical structures along the needle passage. The well-known pain-sensitive tissues related to the spine are the skin, ligaments, periosteum, dura mater, and paravertebral muscles [31]. The midline technique penetrates the supraspinous and interspinous ligaments, ligament flavum, and dura mater to reach the intrathecal space, contrary to the paramedian approach, which enters only the ligamentum flavum and dura mater without ligament puncture [4]. This ligament trauma induced by the midline approach may increase pain intensity after spinal anesthesia. Another proposed factor contributing to LBP after spinal anesthesia involves excessive stretching and straining of spinal ligaments [30]. Hence, when employing the paramedian approach, which involves less bending of the back during the procedure, this technique might mitigate an excessive stretching of spinal ligaments. This reduction in stretching, theoretically, could lead to a potentially lower occurrence of LBP following spinal anesthesia. 

In general, the paramedian technique has technical advantages over the midline approach in patients with degenerative changes in the spinal structures or who have difficulty maintaining a fully flexed position [32]. As the entry point of the paramedian approach is 1 cm inferolateral to the spinous process, bony obstacles can be avoided, thereby increasing the success rate of dural puncture [33]. Most studies included in our meta-analysis investigated parturients undergoing cesarean section. Most full-term parturients find it challenging to assume an effective posture for spinal anesthesia; thus, the intervertebral space may not be sufficiently widened. This may explain the higher success rate observed with the paramedian approach compared to that of the midline technique.

The risk of bias in the included studies was mostly rated as “some concerns.” This implies that the results from these studies might contain plausible biases, warranting caution, particularly regarding the interpretation and reliability of the outcomes. However, the most common reasons for “some concerns” were due to inadequate information on concealment and insufficient details regarding pre-specified analysis plans. However, considering the characteristics of the investigated outcomes, PDPH and LBP are determined based on patients’ subjective experiences. Therefore, the concealment and the alignment between pre-specified analysis plans and the actual conducted analysis might have minimal impact on these outcomes. Additionally, for the outcome of a first-attempt success rate, which is not heavily influenced by concealment or pre-specified analysis plans, the clinical significance of bias in this outcome is perceived to be low.

Our meta-analysis had several limitations. First, most included studies were conducted on relatively young parturients who underwent cesarean section, possibly limiting the result’s generalizability. In particular, regarding our primary outcome, PDPH, Al-Hashel et al. [34] indicated a higher occurrence in younger patients and more frequently in females than males. As the majority of studies included in our analysis focused on young parturients undergoing cesarean delivery, the incidence of PDPH itself might be comparatively higher than in other population groups. Therefore, expecting equivalent effects across different population groups solely based on these studies might be challenging. It underscores the need for additional research to ensure the generalizability of these findings across diverse populations. Second, subgroup analyses according to needle type and size could not be performed owing to insufficient studies for subgrouping and data synthesis. The cutting type of the needle is a suggested risk factor for PDPH compared to the pencil-point needle [35]. This underscores the need for more clinical studies to investigate the impact of needle type on PDPH in various needle advancement methods, like paramedian and midline approaches. Third, we limited our literature search to PubMed, EMBASE, CENTRAL, Scopus, and Web of Science databases. Therefore, studies retrieved from other databases may have been excluded. Fourth, the I^2^ value for the PDPH incidence was relatively high at 53%. This could potentially stem from the diversity in the types of spinal needles used across the included studies. Previous reports [26,36] suggest that smaller needle diameters and atraumatic needles are associated with lower PDPH incidence compared to larger needle diameters and traumatic needles. In our meta-analysis, the included studies encompassed needle diameters ranging from 25 G to 27 G, comprising both atraumatic and traumatic needle types. This mixture of needle diameters and types might contribute to the observed moderate degree of heterogeneity. Fifth, this study did not encompass research on spinal anesthesia utilizing ultrasound guidance. The use of ultrasound is increasingly prevalent across various fields, and there is growing interest in ultrasound-guided spinal anesthesia [37,38]. However, as of now, the routine use of ultrasound in performing spinal anesthesia is not as widespread as the conventional landmark-based technique. Given that the primary aim of this study was to investigate the differences in approaches within the commonly practiced conventional landmark technique, we purposefully excluded studies with ultrasound-based methodologies. It is believed that future meta-analyses could provide valuable insights into a more comprehensive understanding of the landmark-based and ultrasound-guided techniques, as well as their respective advantages and limitations. Finally, most studies were assessed to have “some concerns” for overall bias. Therefore, these findings should be interpreted circumspectly.

## 5. Conclusions

In conclusion, spinal anesthesia using the paramedian approach resulted in lower incidences of both PDPH and LBP. The paramedian approach also has a higher success rate at first attempt than the midline approach. However, clinical implications for the general surgical population should be cautiously interpreted, and more well-planned clinical trials are required to verify and generalize the outcomes. In particular, the majority of studies included in our meta-analysis conducted experiments primarily on young women. However, considering that PDPH occurs more frequently in young women and that the paramedian approach might offer advantages over the midline approach in patients with spinal degenerative changes or those unable to maintain a fully flexed position, different effects might be observed in populations comprising older individuals or males. Additionally, patients undergoing hip fracture surgery, where maintaining a fully flexed position might be challenging, could exhibit varied outcomes. Hence, for practical application in these surgical and age groups, further research would be essential.

## Figures and Tables

**Table 1:** Summary of characteristics of included studies and outcome data.

Source	Number of Participants		Type of Surgery	Type and Gauge of Needle	Events of PDPH		Events of Low Back Pain		Number of Success on First Attempt	
	Paramedian	Median			Paramedian	Median	Paramedian	Median	Paramedian	Median
Bansal, 2018 [8]	100	100	Cesarean delivery	23 G Quincke	1	5	NI	NI	80	75
Firdous, 2016 [16]	60	60	Cesarean delivery	25 G pencil-point	1	3	NI	NI	NI	NI
Karami, 2021 [10]	68	68	Cesarean delivery	25 G Quincke	7	7	NI	NI	NI	NI
Kumari, 2021 [17]	50	50	Cesarean delivery	Sprotte *	4	8	2	3	47	43
Lee, 2020 [25]	50	50	Several types **	25 G pencil-point	NI	NI	8	18	51	52
Mahrous, 2021 [18]	135	149	Cesarean delivery	25 G Quincke	NI	NI	NI	NI	131	141
Mohamed, 2021 [19]	30	30	Cesarean delivery	NI	5	22	6	8	29	28
Mohammad, 2008 [24]	50	50	Lower abdominal surgery	25 G Quincke	NI	NI	27	43	36	17
Montasser, 2015 [20]	200	200	Cesarean delivery	27 G Quincke	4	6	NI	NI	NI	NI
	200	200	Cesarean delivery	27 G Whitacre	0	3	NI	NI	NI	NI
Nisar, 2016 [21]	50	50	Cesarean delivery	25 G ^†^	4	2	NI	NI	NI	NI
Rabinowitz, 2007 [22]	20	20	Hip fracture surgery	19 G Tuohy	NI	NI	NI	NI	17	9
Sheibany, 2014 [11]	70	70	Cesarean delivery	25 G ^†^	24	27	19	49	NI	NI
Singh, 2018 [23]	50	50	Lower abdominal surgery	25 G ^†^	2	10	1	5	NI	NI

PDPH, post-dural puncture headache; NI, no information. * Information on gauge of needle was not provided. ** Several types of surgery included urological, orthopedic, gynecological and general surgeries. ^†^ Type of needle was not specified.

## Data Availability

No new data were created or analyzed in this study. Data sharing is not applicable to this article.

## Supplementary Materials

The following supporting information can be downloaded at: https://www.mdpi.com/article/10.3390/medicina60010178/s1, Table S1: Search strategy for each database; Table S2: Level of Certainty for each outcome.
