# Supplementary material for: Comparison of the Effect of Landmark-Based Midline and Paramedian Approaches on Spinal Anesthesia-Related Complications in Adult Patients: A Meta-Analysis of Randomized Controlled Trials
Source: Medicina (Kaunas). 2024 Jan 19;60(1):178. doi: 10.3390/medicina60010178 (PMC10819311; doi:10.3390/medicina60010178)
Supplement: Supplementary file 1 [file medicina-60-00178-s001.zip › medicina-2810937-supplementary.pdf]

Supplementary Files

Supplementary Table S1. Search strategy for each database. (20221219)

Database	Order	Keywords	Results
PubMed	#1	((((median[Title]) OR (midline[Title])) OR (para-median[Title])) OR (paramedian[Title])) OR (lateral[Title])	83576
	#2	((((spinal[Title]) OR (intrathecal[Title])) OR (intra-thecal[Title])) OR (sub-arachnoid[Title])) OR (subarachnoid[Title])	167198
	#3	((anesthesia[Title]) OR (anaesthesia[Title])) OR (block[Title])	153241
	#4	#1 AND #2	1506
	#5	#3 AND #4	99
EMBASE	#1	median:ti OR midline:ti OR 'para median':ti OR paramedian:ti OR lateral:ti	97514
	#2	spinal:ti OR intrathecal:ti OR 'intra thecal':ti OR 'sub arachnoid':ti OR subarachnoid:ti	206120
	#3	anesthesia:ti OR anaesthesia:ti OR block:ti	185487
	#4	#1 AND #2	1761
	#5	#3 AND #4	124
CENTRAL	#1	(median):ti OR (midline):ti OR (para-median):ti OR (paramedian):ti OR (lateral):ti	5264
	#2	(spinal):ti OR (intrathecal):ti OR (intra-thecal):ti OR (subarachnoid):ti OR (sub-arachnoid):ti	17373
	#3	(anesthesia):ti OR (anaesthesia):ti OR (block):ti	38331
	#4	#1 AND #2	181
	#5	#3 AND #4	130
SCOPUS	#1	(TITLE (median) OR TITLE (midline) OR TITLE (para-median) OR TITLE (paramedian) OR TITLE (lateral))	139599
	#2	(TITLE (spinal) OR TITLE (intrathecal) OR TITLE (intra-thecal) OR TITLE (subarachnoid) OR TITLE (sub-arachnoid))	191984
	#3	(TITLE (anesthesia) OR TITLE (anaesthesia) OR TITLE (block))	305141
	#4	#1 AND #2	1635
	#5	#3 AND #4	117
Web of Science	#1	median (Title) or midline (Title) or para-median (Title) or paramedian (Title) or lateral (Title)	124197
	#2	spinal (Title) or intrathecal (Title) or intra-thecal (Title) or subarachnoid (Title) or sub-arachnoid (Title)	180611
	#3	anesthesia (Title) or anaesthesia (Title) or block (Title)	284865
	#4	#1 AND #2	1566
	#5	#3 AND #4	94

Supplementary Table S2 Level of Certainty for each outcome

Certainty assessment							No. of patients		Effect		Certainty
**No. of studies**	**Study design**	**Risk of bias**	**Inconsistency**	**Indirectness**	**Imprecision**	**Other considerations**	**Paramedian approach**	**Median approach**	**Relative (95% CI)**	**Absolute (95% CI)**	
10	randomised trials	not serious	not serious	not serious	not serious	none	52/878 (5.9%)	93/878 (10.6%)	**OR 0.43** (0.22 to 0.83)	**57 fewer per 1,000** (from 81 fewer to 15 fewer)	⊕⊕⊕⊕ High
6	randomised trials	not serious	not serious	not serious	not serious	none	63/300 (21.0%)	126/300 (42.0%)	**OR 0.27** (0.16 to 0.44)	**256 fewer per 1,000** (from 316 fewer to 178 fewer)	⊕⊕⊕⊕ High
7	randomised trials	serious	not serious	not serious	not serious	none	391/445 (87.9%)	365/463 (78.8%)	**OR 2.30** (1.36 to 3.87)	**107 more per 1,000** (from 47 more to 147 more)	⊕⊕⊕◯ Moderate

The level of evidence was assessed by the GRADE method, from very low ⊕⊖⊖⊖ to high ⊕⊕⊕⊕. No.: number; CI: confidence interval; OR: odds ratio.
